# Influence of Extracellular Matrix Components on the Expression of Integrins and Regeneration of Adult Retinal Ganglion Cells

**DOI:** 10.1371/journal.pone.0125250

**Published:** 2015-05-27

**Authors:** Elena Vecino, Janosch P. Heller, Patricia Veiga-Crespo, Keith R. Martin, James W. Fawcett

**Affiliations:** 1 Dept. of Cell Biology and Histology, University of the Basque Country, UPV/EHU, Leioa, Vizcaya, Spain; 2 John van Geest Centre for Brain Repair, Department of Clinical Neurosciences, University of Cambridge, Cambridge, United Kingdom; 3 Welcome Trust—MRC Cambridge Stem Cell Institute, University of Cambridge, Cambridge, United Kingdom; NIH/NEI, UNITED STATES

## Abstract

**Purpose:**

Retinal ganglion cells (RGCs) are exposed to injury in a variety of optic nerve diseases including glaucoma. However, not all cells respond in the same way to damage and the capacity of individual RGCs to survive or regenerate is variable. In order to elucidate factors that may be important for RGC survival and regeneration we have focussed on the extracellular matrix (ECM) and RGC integrin expression. Our specific questions were: (1) Do adult RGCs express particular sets of integrins *in vitro* and *in vivo*? (2) Can the nature of the ECM influence the expression of different integrins? (3) Can the nature of the ECM affect the survival of the cells and the length or branching complexity of their neurites?

**Methods:**

Primary RGC cultures from adult rat retina were placed on glass coverslips treated with different substrates: Poly-L-Lysine (PL), or PL plus laminin (L), collagen I (CI), collagen IV (CIV) or fibronectin (F). After 10 days in culture, we performed double immunostaining with an antibody against βIII-Tubulin to identify the RGCs, and antibodies against the integrin subunits: αV, α1, α3, α5, β1 or β3. The number of adhering and surviving cells, the number and length of the neurites and the expression of the integrin subunits on the different substrates were analysed.

**Results:**

PL and L were associated with the greatest survival of RGCs while CI provided the least favourable conditions. The type of substrate affected the number and length of neurites. L stimulated the longest growth. We found at least three different types of RGCs in terms of their capacity to regenerate and extend neurites. The different combinations of integrins expressed by the cells growing on different substrata suggest that RGCs expressed predominantly α1β1 or α3β1 on L, α1β1 on CI and CIV, and α5β3 on F. The activity of the integrins was demonstrated by the phosphorylation of focal adhesion kinase (FAK).

**Conclusions:**

Adult rat RGCs can survive and grow in the presence of different ECM tested. Further studies should be done to elucidate the different molecular characteristics of the RGCs subtypes in order to understand the possible different sensitivity of different RGCs to damage in diseases like glaucoma in which not all RGCs die at the same time.

## Introduction

Retinal ganglion cells (RGCs) are responsible for the communication between the eye and the brain, extending long axons along the visual pathway through a variety of extracellular matrix (ECM) molecules. After axotomy, the regenerative capacity of the RGCs is limited *in vivo* [1] and they are the first retinal cells to die in neurodegenerative eye diseases such as glaucoma [2]. However, not all cells die at the same time some being more resistant to cell death than others [3]. Thus, large RGCs seem to be more resistant to axotomy in adult rats [1] while they appear to be the most susceptible to death in glaucoma [4], [5] or *in vitro* when cultured with high doses of glutamate [6].

During development, RGCs extend their axons along specific pathways to establish highly ordered innervation patterns. The interaction of neuronal growth cones with their microenvironments promotes growth and directs axons to their targets. Neurites show an ability to discriminate between different substrates, and thus elongate selectively on different surfaces. [7]. Moreover, these surface preferences change between developmental and adult stages [8,9]. Nevertheless, RGCs have to preserve their capacity to interact with different substrata. Thus, in the adult, the cell body and dendrites are in direct contact with Müller cells and astrocytes, and they are also in contact with laminin and collagen in the inner limiting membrane. After leaving the retina via the optic nerve, axons are in contact with oligodendrocytes, astrocytes, and ECM molecules such as collagens. Finally in the brain, additional ECM components surround the axonal terminals, which not only show a change in composition but also a change in texture [10]. Very little is known about the subcellular distribution of receptors within these complex cells. However, Müller glia cells are polarized and while their end feet promote axonal growth their somas support dendritic development of RGCs [11].

Both neuronal survival and axon growth depend on adhesion and signalling from cell surface receptors, but survival and growth signalling differs and neuronal survival alone is not sufficient to elicit robust axon growth [12]. Previous studies by our group demonstrated that adult RGCs growing *in vitro* could respond to the same neurotrophic cues found *in vivo* [13]. Moreover, adult RGCs growing *in vitro* have different survival rates and neurite branching capacities depending on the substratum or the conditioned media in which they grow [14]. This demonstrates that within the retina, different RGCs coexist with differing morphology and molecular characteristics. RGCs have been morphologically classified in a large number of species based mainly on soma size and dendritic field dimensions, dendritic field diameter and level of dendritic arborisation. The dendritic trees of the RGCs determine the position, size and shape of the receptive field. In certain species, this analysis has been associated with functional data demonstrating that different RGC classes project to different targets, which control different visual functions [15]. RGCs therefore comprise several classes, with distinct anatomical and physiological properties, but little is known about the molecular characteristics of the different RGC classes. In the present study we demonstrate that different RGC types respond differently to different substrata.

Integrins are a family of cell surface receptors that are responsible for cell adhesion to ECM proteins. They connect the extracellular environment with the intracellular cytoskeleton, and they are responsible for activation of many intracellular signalling pathways [16]. All integrins are non-covalently linked, heterodimeric molecules containing two subunits, α and β. Each αβ combination has its own specificity and signalling properties. Most integrins recognize several ECM proteins. Conversely, individual matrix proteins, such as fibronectin, laminins, collagens, and vitronectin bind to several integrins. The extracellular binding activity of integrins is regulated from the inside of the cell (inside-out signalling), while the binding of the ECM elicits signals that are transmitted into the cell (outside-in signalling) [17]. In mammalian genomes, to date 24 different αβ combinations have been identified at the protein level. Although some subunits appear only in a single heterodimer, twelve integrins contain the α1 subunit and five contain αV [18].

Since ECM molecules can promote axonal growth, and different RGCs types grow in culture at different rates, we investigated the ability of various ECM molecules to induce axon growth from purified adult RGCs in cell culture to answer the following questions: Do different integrins and substrates correlate with RGC neurite length or branching rate? Can we classify the different cell types by substrate preference or integrin expression?

We grew primary adult RGCs on five different ECM substrata, and we analysed the expression of six different integrin subunits implicated in their link with the studied substrata. We analysed the relationship between substrates and survival of RGCs, branching complexity and length of their neurites. Moreover, the relation between the integrin expression profile of RGCs and the substrates in which the cells were growing was also analysed and compared to integrin expression *in vivo*.

## Materials and Methods

### Animals

All animal experimentation adhered to the ARVO Statement for the Use of Animals in Ophthalmic and Vision Research and to the United Kingdom Animals (Scientific Procedure) Act of 1986. Adult female Sprague-Dawley rats (250–300g) were obtained from Harlan Laboratories and were housed under a 12 hour light-dark cycle with access to food and water ad libitum. All methods were approved by the University of Cambridge Animal Ethics Committee and are in conformity with the “Guiding Principles for research Involving Animals and Human´s as adopted by the American Physiological Society. Animals were humanely sacrificed by exposure to CO_2_.

### RGC cultures

Retinae were dissected and dissociated enzymatically to obtain a mixed suspension of retinal cells using the Papain Dissociation Kit (Worthington Biochem, New Jersey, USA) according to the manufacturer's instructions. Dissociated retinal cells were plated on 13 mm diameter coverslips (VWR, Lutterworth UK) pre-coated with the different substrates in 24 well plates (Fisher Scientific, Loughborough) at 10^5^ viable cells per well (determined by trypan blue test). Glass coverslips were coated with the following substrata: Poly-L-lysine (PL) (Sigma, P4832 at 20 μg/ml) only or PL followed by laminin (L) (Sigma, L2020, 10 μg/ml), collagen I (CI) (Sigma C8919, 10 μg/ml), collagen IV (CIV) (Sigma C5533, 10 μg/ml) or fibronectin (F) (Sigma, F1141, 10 μg/ml). All coverslips were coated with PL overnight in the incubator at 37°C before the ECM molecules were added for at least two hours at room temperature. All coating substrata were diluted in PBS. After each coating step, the coverslips were washed twice with PBS.

Retinal cells were cultured in B27-supplemented Neurobasal-A medium (Invitrogen) containing L-glutamine and gentamicin for 6 days at 37°C in a humidified atmosphere containing 5% CO_2_. Medium was changed every 3 days and no growth factors were added to the culture medium. Three independent experiments were repeated. In each experiment the cells were cultured on the 5 different substrates and 6 coverslips were used per substratum so we could test the anti-integrin antibodies (Table 1).

**Table 1:** Antibodies used in the present study indicating source, manufacturer, catalogue number and dilution.

Antibody	Source	Manufacturer	Ref.	Dilution
**α1**	Rabbit	Millipore	AB1934	1:500
**α3**	Rabbit	Santa Cruz	SC7019	1:500
**α5**	Rabbit	Chemicon	CH1928	1:500
**αv**	Rabbit	Millipore	AB1930	1:800
**β1**	Rabbit	Millipore	AB1952	1:500
**β3**	Rabbit	Millipore	AB2984	1:500
**Colagen I**	Rabbit	Sigma	SAB4500362	1:200
**Colagen IV**	Rabbit	Sigma	SAB4500369	1:200
**Fibronectin**	Rabbit	Sigma	F3648	1:500
**Laminin**	Rabbit	Sigma	L9393	1:50
**Tubulin III-β**	Mouse	Promega	G712A	1:2000
**FAK (pY-397)**	Rabbit	Biosource	44-626G	1:100
**Biotinilated**	Rabbit	Vector	BA100	1:1000
**Streptabidin-Biotin Alexa Fluor 568**	Anti-Biotin	Vector	S511226	1:500
**Streptabidin- Alexa Fl 488**	Donkey anti-Mouse	Invitrogen	Lot811493	1:1000
**Alexa 488**	Goat anti-rabbit	Life Tech	A-11034	1:1000
**Alexa 555**	Goat anti-mouse	Life Tech	A-21424	1:1000

### Immunohistochemistry on tissue sections

Animals were anesthetized with an intraperitoneal injection of a cocktail of ketamine 60 mg/kg and xylazine 7.5 mg/kg prior to the intra-cardial perfusion with saline (0,9% NaCl) followed by 4% paraformaldehyde (PFA)/0.1 M PBS. Eyes and optic nerves were removed and post-fixed by immersion overnight in cold 4% PFA. Tissue was then washed with PBS and transferred to 30% sucrose solution (overnight at 4°C) for cryoprotection and embedded in OCT mounting medium (Raymond A. Lamb, Eastbourne, UK). Serial sections, 14 μm thick, were cut with a cryostat, thaw-mounted onto glass slides (Superfrost plus, VWR, Lutterworth, UK) and stored at −20°C until further use. After blocking, rehydrated eye sections were incubated with primary antibody (in blocking solution; 4% goat serum in PBS; 0.3%Triton) overnight at 4°C: mouse anti-βIII tubulin antibody and rabbit anti- either (laminin, collagen I, collagen IV, fibronectin, α1, α3, α5, αV, β1, β3 integrin) at the dilution indicated in Table 1. The following day, slides were washed and secondary antibodies (Alexa 488 goat anti-rabbit; 1:1000 and Alexa 555 goat anti-mouse; 1:1000, Life Tech were used for the ECM and βIII tubulin antibodies; streptavidin-biotin (see below) was used to amplify the integrin antibody signal) were applied for 1 hour at room temperature in blocking solution. After washing, slides were mounted in Fluorsave (Calbiochem/Merck Chemicals, Beeston, UK).

### Immunocytochemistry on cells

The cells were fixed in ice-cold methanol for 5 minutes and then washed 4 times in PBS followed by a blocking incubation with skimmed milk (2 g/ml) in PBS for 30 minutes. After blocking, the coverslips were incubated with primary antibody diluted in the blocking solution overnight at 4°C: mouse anti-βIII tubulin antibody (1:2000, Promega) combined with one of the anti-integrin antibodies (α1, α3, α5, αV, β1, β3) at the dilution indicated in Table 1.

The following day, the coverslips were washed and incubated with a secondary biotinylated anti-rabbit antibody, (1:1000, Vector) for 30 minutes, washed three times and followed by 45 minutes incubation with Streptavidin-Alexa Fluor 568 (1:500, Vector) to stain the different integrins and Alexa Fluor donkey anti-mouse 488 (1:500, Invitrogen) to stain the βIII-tubulin positive RGCs. Coverslips were then washed in PBS, followed by nuclear counterstaining with 4,6-diamidino-2-phenylindole (1:10,000, DAPI, Sigma). After a final wash in PBS, coverslips were mounted in Fluorsave (Calbiochem/Merck Chemicals, Beeston, UK).

### Quantification of RGC survival/neurite complexity and length

The analysis of RGC morphology was done by taking a mosaic of pictures at 10X (Pathway 855, Becton Dickinson). The number of RGCs, the number of neurites growing from each RGC (complexity), and the length of each neurite were measured and the cells were classified by length of the longest neurite in at least 3 wells/experimental condition in a total of three independent experiments.

For the retina and optic nerve, at least 3 sections were analysed from each of 3 rats for the analysis. To measure the *in vitro* expression of integrins, at least 3 coverslips per integrin and per substrate were analysed in at least three independent experiments. Analysis was performed using AttoVision (Beckton Dickinson)

### Statistical analysis

The influence of the different substrates was analysed statistically taking the following parameters into account: (1) the numbers of cells surviving on the different substrata; (2) the complexity of processes, calculated as described above (3) stimulation of axon growth by measuring the length of the longest neurite.

For the complexity parameter, cells were classified as having a **low** level of complexity when they did not have any neurite; a **medium** level of complexity when the RGCs had between 1 and 3 neurites; and a **high** level complexity when the cells showed 4 or more neurites.

Neurite length, measured from the longest neurite, was also classified into three groups. Group **short** included cells with a longest neurite <62 μm; group **medium** length comprised cells with the longest neurite between 62 μm and 248 μm; and group **long** contained RGCs with neurites longer than 248 μm.

Statistical analyses were carried out using IBM SPSS Statistics software v. 21.0. The influence of substrates on the above-mentioned parameters was measured by one-way ANOVA (p < 0.05) with Bonferroni and Scheffe post-hoc test (p < 0.05). The homogeneity of the variance was assayed by Levene´s test (p < 0.05). When the homogeneity of the variance was violated, the Welch ANOVA and the post-hoc test of T2 of Tamhane and T3 of Dunnet were applied.

## Results

### Cell survival varies with substrate

A total of 1900 cells was analysed throughout all experiments.

We analysed their survival on five substrates: (PL) Poly-L-Lysine, L (laminin), C1 (type 1 Collagen), CIV (Type IV Collagen), F (Fibronectin).

We used βIII-tubulin to visualise RGCs because it is a phenotypic marker for RGC somata and their processes, which was found to specifically correlate to DiI retrogradely labelled RGCs, suggesting that it is a reliable indicator of the number of surviving RGCs [19].

**Fig 1:**
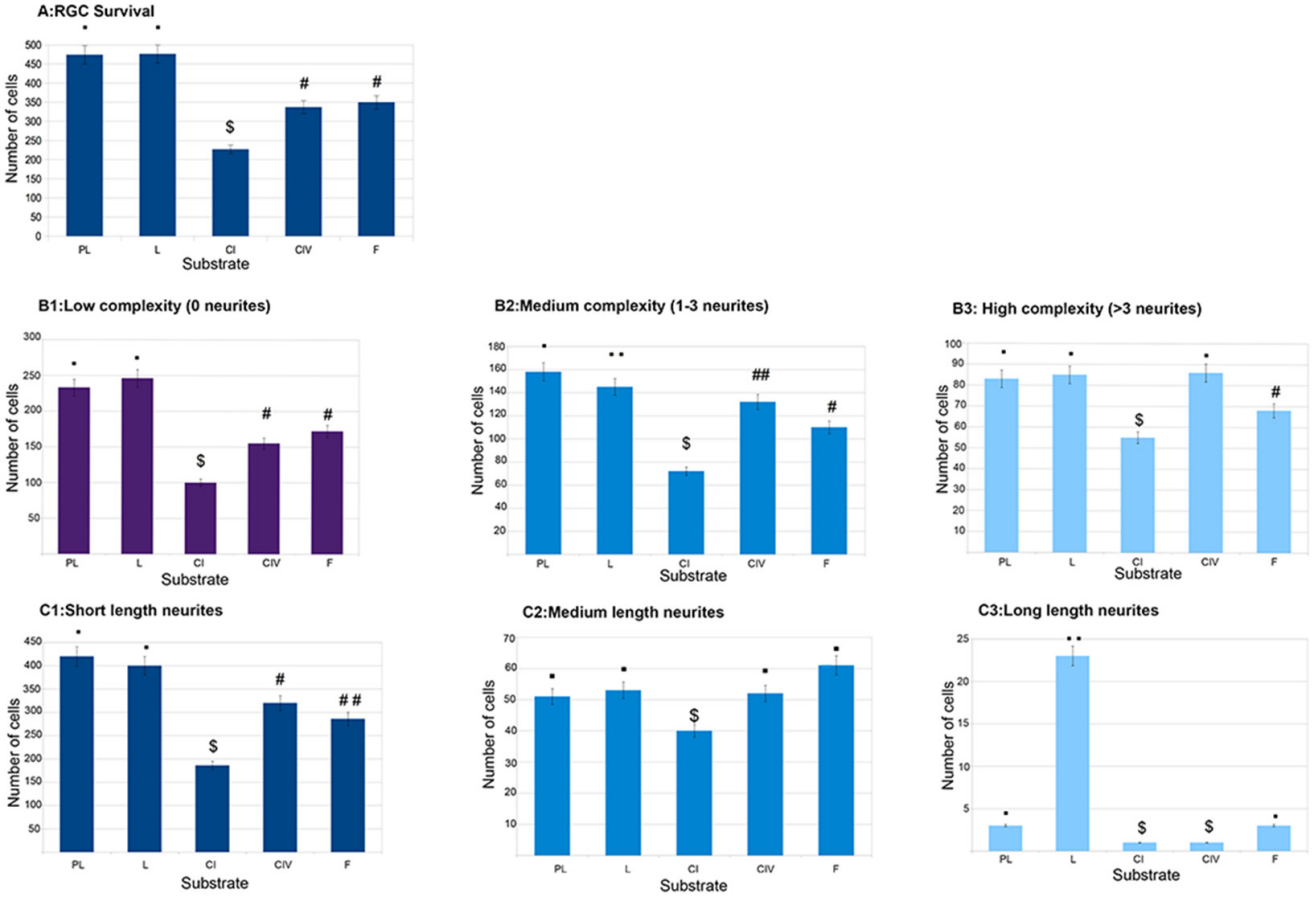
Influence of the substrata and cell distribution. In all cases the statistical difference was (p<0.05). **A: Influence of the substrata on cell survival.** The influence of the substrate on the number of observed cells PL and L (•) show significant differences compared to the other substrates; CI ($) shows significant differences in the number of cells compared to the other substrates and CIV and F (#) have significant differences compared to the other substrates **B: Distribution of RGC complexity (number of neurites per cell). B1: Low level of complexity.** Significant differences were found for PL and L (•) against the rest of the substrates; CI ($) against the rest of substrates and CIV and F (#) with the rest of the substrates. **B2: Medium level of complexity.** Significant differences were found for each substrate compared to the other substrates. B3: High level of complexity. Significant differences were found for PL, L and CIV (•) against CI and F; CI ($) and F (#) was significantly different to all other substrates. **C: Distribution of RGC neurites length between the different substrates. C1: Short length** RGCs growing in PL and L (•) are significantly different to the other substrates; and the rest of the RGCs growing in CI ($), CIV (#), or F (##) each was different to the other substrates. **C2 Medium length.** CI ($) was significantly different to the other substrates, but there was no difference between them PL, L, CIV and F (•). **C3 Long length** neurites were more frequent when RGCs were plated on L (••) and this difference was significant compared to the other substrates. PL and F (•) were the second substrate in which long neurites were present; CI and CIV had significantly fewer long neurites.

The mean number of surviving attached cells after ten days on PL and L was higher than the number of cells surviving on the other 3 substrates (PL 474 ± 59, 24,9% and L 476 ± 57, 25,1%). The lowest number of RGCs was present on CI (227 ± 17, 11,9%), while CIV and F had similar numbers of cells (337 ± 29 and 350 ± 34, respectively). These differences were statistically significant (p<0.05). In conclusion, PL and L favoured the survival of RGCs (Fig 1).

### Relationship between substrate and complexity

Next, we analysed the number of neurites growing from each cell. For each substrate we counted the number and the percentage of cells that had low (0 neurites), medium (1–3 neurites) or high (>3 neurites) complexity (Table 2A and 2B, and Fig 1). The type of cells most commonly found in the cultures independent of all substrates, were cells with low complexity (0 neurites). On the other hand, only few cells had high complexity (3 or more neurites) within each substrate group.

**Table 2:** Relationship between the substrates and the number of emitted neurites.

A: Number of RGC	Level of Complexity		
Substrate	Low	Medium	High
	0 neurites	1–3 neurites	> 3 neurites
**PL**	233 ± 35	158 ± 36	83 ± 32
**L**	246 ± 38	145 ± 35	85 ± 38
**CI**	100 ± 18	72 ± 25	55 ± 21
**CIV**	155 ± 29	132 ± 26	86 ± 26
**F**	172 ± 28	110 ± 28	68 ± 26
**B: % Inside the total analysed cells**	**Level of Complexity**		
**Substrate**	**Low**	**Medium**	**High**
**PL**	12,3%	8,3%	4,4%
**L**	12,9%	7,6%	4,5%
**CI**	5,3%	3,8%	2, 9%
**CIV**	9,1%	5,8%	3,6%
**F**	47,7%	32,5%	19, 8%

This relation was named complexity, and was considered in three levels attending to the number of neurites emitted per cell: Low (0 neurites), Medium (1–3 neurites) and High (>3 neurites).

When we looked at each substrate individually (Table 2A), we found that there was a higher proportion of cells with high complexity on CI and CIV substrata, although survival of cells was less than 50% on C1 compared to PLL or L (Fig 1).

These differences were significant by ANOVA analysis and post-hoc analysis showed significant differences between the substrata and the different complexity categories (p<0.05) (Fig 1).

Taken together, PL and L were the best substrata to promote the attachment and the growth of cells while CI and CIV promoted higher complexity (Table 2B).

### Relationship between substrate and the length of the neurites

The majority of RGCs on all substrates had no neurite or very short neurites, and hence belonged to the group that we called short (Table 3, Fig 1). Cells with very long neurites (>248 μm) were uncommon, although some of these cells, mainly on L, had a very long neurite of several thousand micrometres.

**Table 3:** Relationship between the substrates and the length of the longest neurite inside the different substrates.

Inside the different substrates	Length of the longest neurite		
Substrate	Short	Medium	Long
	<62 μm	62–248 μm	> 248 μm
**PL**	420 ± 36	51 ± 30	3 ± 1
**L**	400 ± 37	53 ± 58	23 ± 4
**CI**	186 ± 20	40 ± 22	1
**CIV**	320 ± 28	52 ± 22	1
**F**	286 ± 28	61 ± 25	3 ± 1

Statistical analysis showed the influence of the substrates on the length of the neurites, and the post-hoc analysis revealed the existence of significant differences between the substrates (Table 3 and Fig 1).

The ability of the cells to grow neurites was clearly influenced by the substrate and the ANOVA analysis confirmed significant differences in the values observed between substrates in all the different post-hoc tests applied. Cells with very long neurites were rarely seen except on L. All substrates showed a similar ability to promote the growth of medium length axons (Fig 1). The CI substrate differed from the others in that the surviving RGCs produced many neurites of medium length, but overall survival was lower.

### Distribution of the extracellular matrix in the retina and optic nerve

In order to observe whether the rat retina and optic nerve contained the ECM molecules that we studied, and to assay integrin receptors at the age at which we dissociated cells for the *in vitro* studies, we performed immunohistochemistry in those tissues. The antibodies used for that purpose are detailed in Table 1.

**Fig 2:**
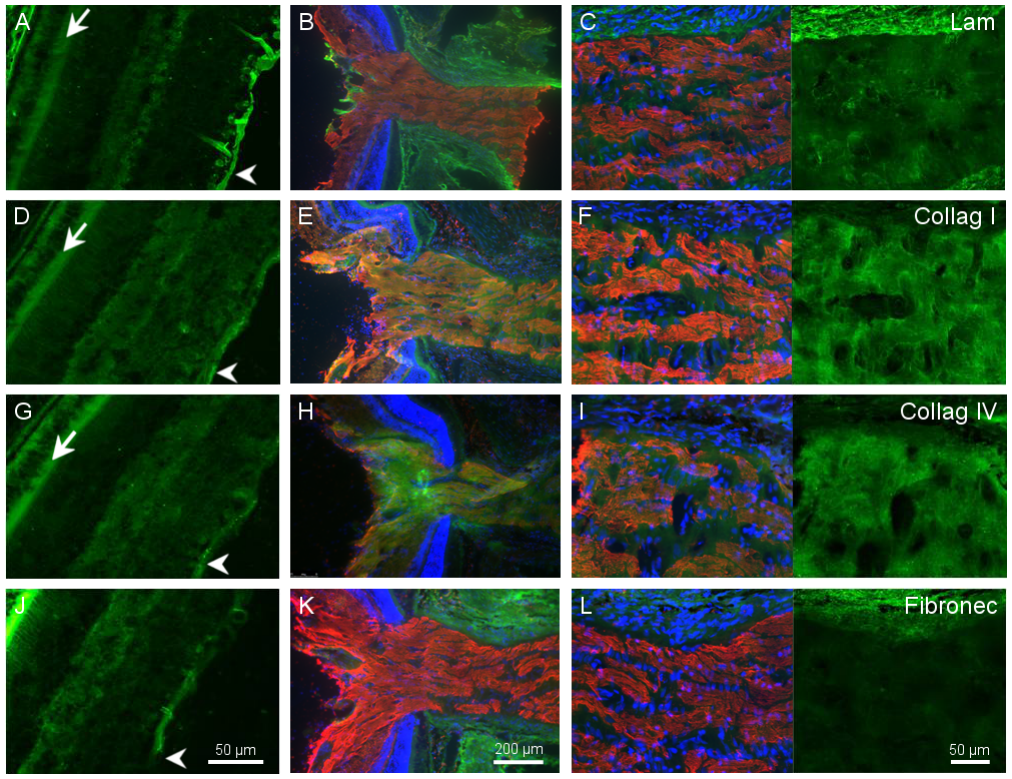
Extracellular matrix distribution in the retina and optic nerve. Distribution of laminin (A,B,C), collagen I (D,E,F), collagen IV (G,H,I) and fibronectin (J,K,L) in green; in the retina (A,D,G,J scale for all pictures in J) the arrowheads point to the inner limiting membrane while arrows point to outer limiting membrane. The optic nerve head (B, E, H, K scale for all pictures in K) and in the optic nerve (C,F,I,L, scale for all pictures in L). βIII-tubulin labelling in the retinal ganglion cell axons is depicted in red, and DAPI labelling to stain cell nuclei is in blue.

Laminin was present within the retina mainly in the inner and outer limiting membranes as well as around the RGCs. Additionally laminin was present in the endothelium of the blood vessels. Within the optic nerve, laminin was present at a low level around the axons and at a higher level in the meninges that surround the optic nerve (Fig 2A–2C).

Collagens I and IV were also present in the limiting membranes. Moreover, in the case of collagen IV, some staining was visible around the RGCs. Both collagens showed bright staining between the axons in the optic nerve (Fig 2D–2I).

Fibronectin was also found in the inner limiting membrane and very intensely in the outer limiting membrane, but was absent within the optic nerve (Fig 2J–2L).

**Fig 3:**
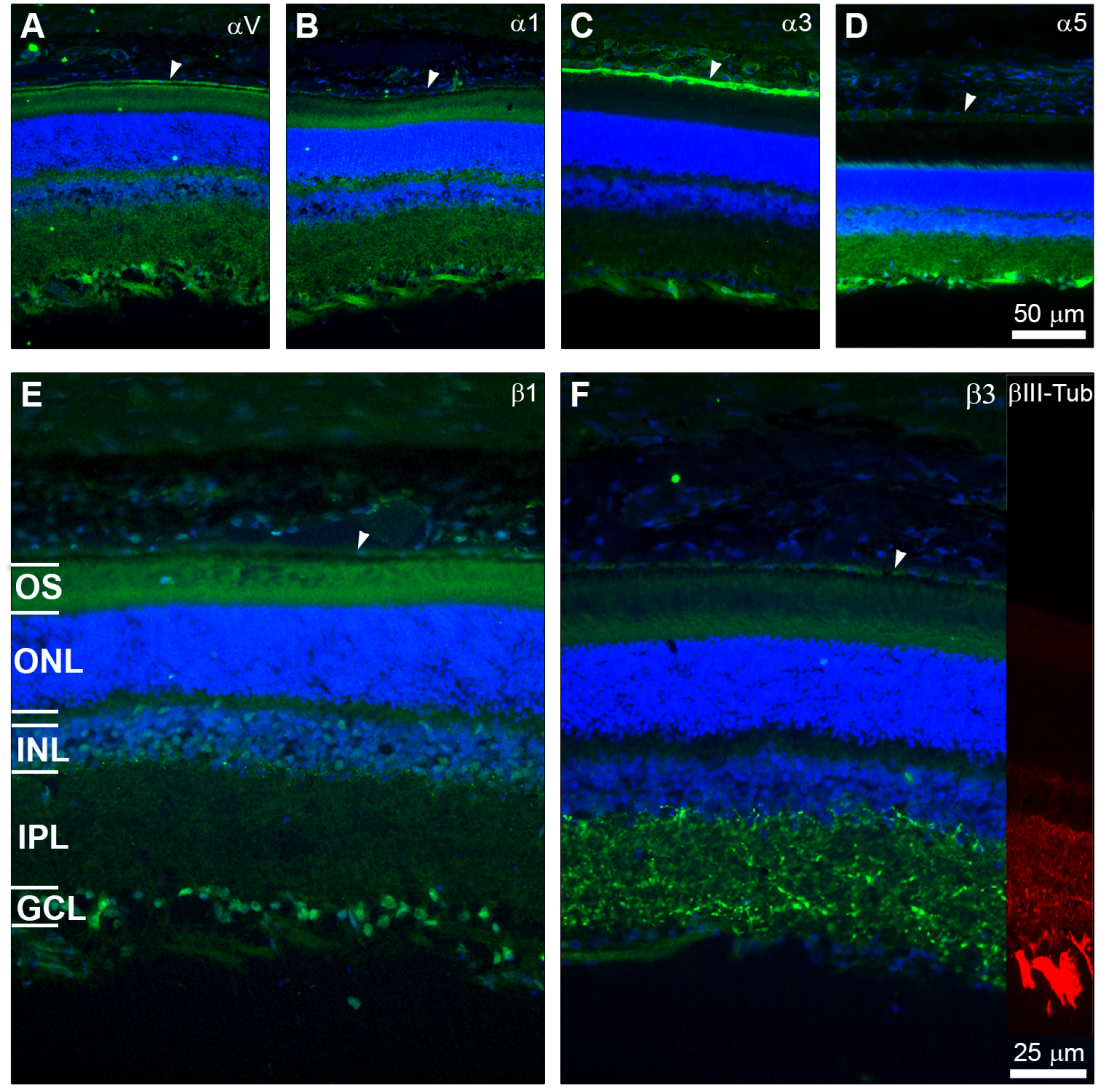
Integrin distribution within the retina. The images show the integrin label in green, βIII-Tubulin in red and DAPI staining in blue. Distribution of αV, α1, α3, α5 in A, B,C,D (scale bar in D for all α) and labelling for β1 and β3 in E, F (scale bar in F for both β). Arrowheads point to the retinal pigment epithelium. The α integrins and β1 were mainly located in the retinal ganglion cell layer, endothelium of the vessels located closed to the RGCs wile β3 was found mainly within the inner plexiform layer. The retina layers are indicated in E. Outer segments (OS), outer nuclear layer (ONL), inner nuclear layer (INL), inner plexiform layer (IPL) and ganglion cell layer (GCL).

The integrin subunits that are implicated in the interaction with the substrata studied were also examined in retina sections. The αV, α1, α3, α5, β1 and β3 integrin subunits were present in the RGC layer with different intensities (Fig 3). α5 and β3 staining was intense in the inner plexiform layer of the retina. In addition to the RGCs, α3 was very intensely stained in retinal pigment epithelial cells and in the endothelia of the blood vessels located in the inner most part of the retina. β1 was found in the RGCs and axons wile β3 was located mainly in the inner plexiform layer (IPL) of the retina (Fig 3).

### Distribution of integrins in the RGCs growing on different substrates

**Fig 4:**
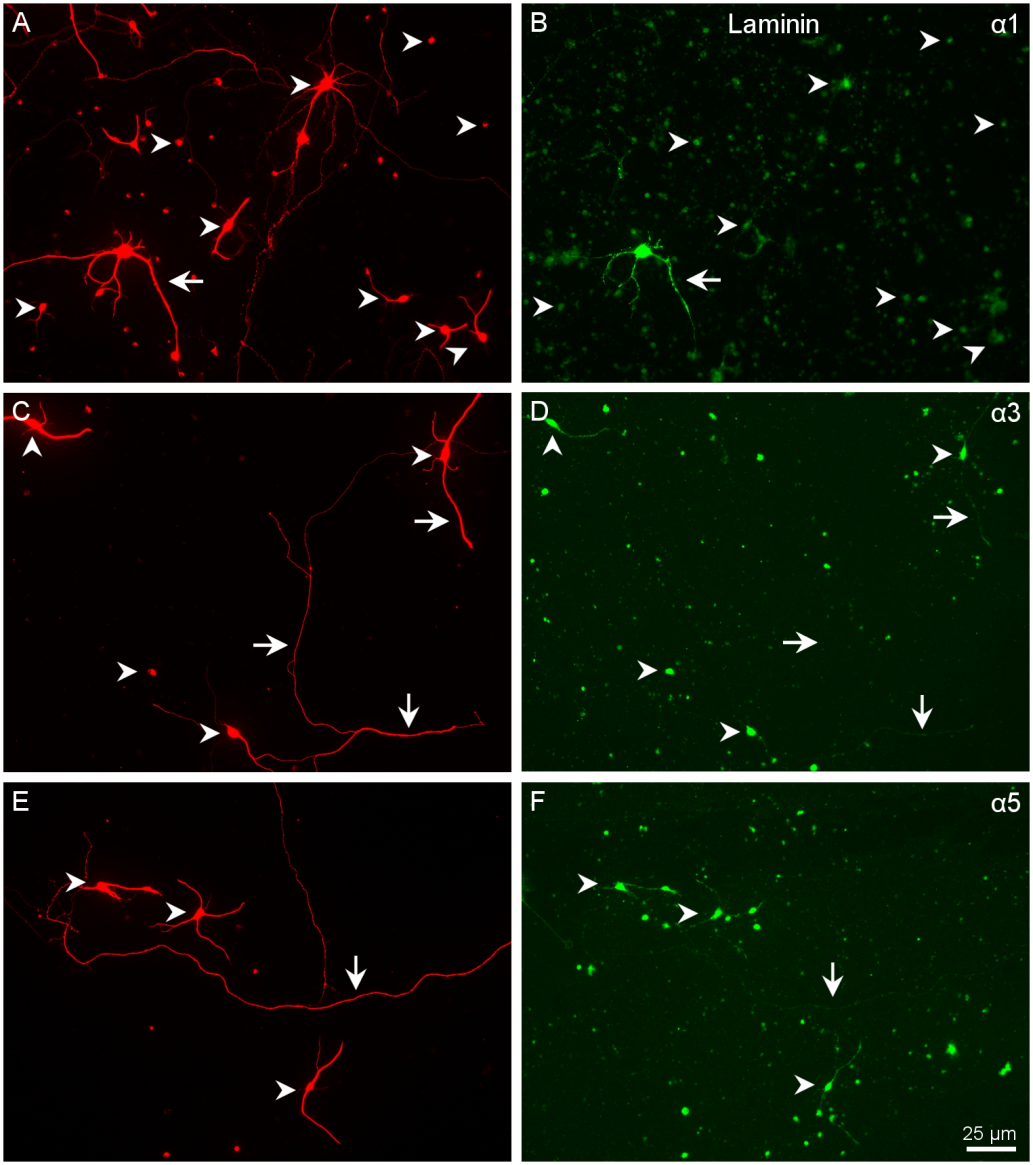
Distribution of α integrins in RGCs growing in laminin. Paired images A-B, C-D, and E-F of RGCs labelled with a β III-tubulin antibody in red (A, C, E) and for different integrins α1 (B), α3 (D) and α5 (F) in green. Arrowheads point to the cell body of the RGCs while the arrows point to the neurites. Note that the central arrow in C of α3 integrin antibody does not label the long neurite pointed out in D. Scale bar for all pictures in F.

**Fig 5:**
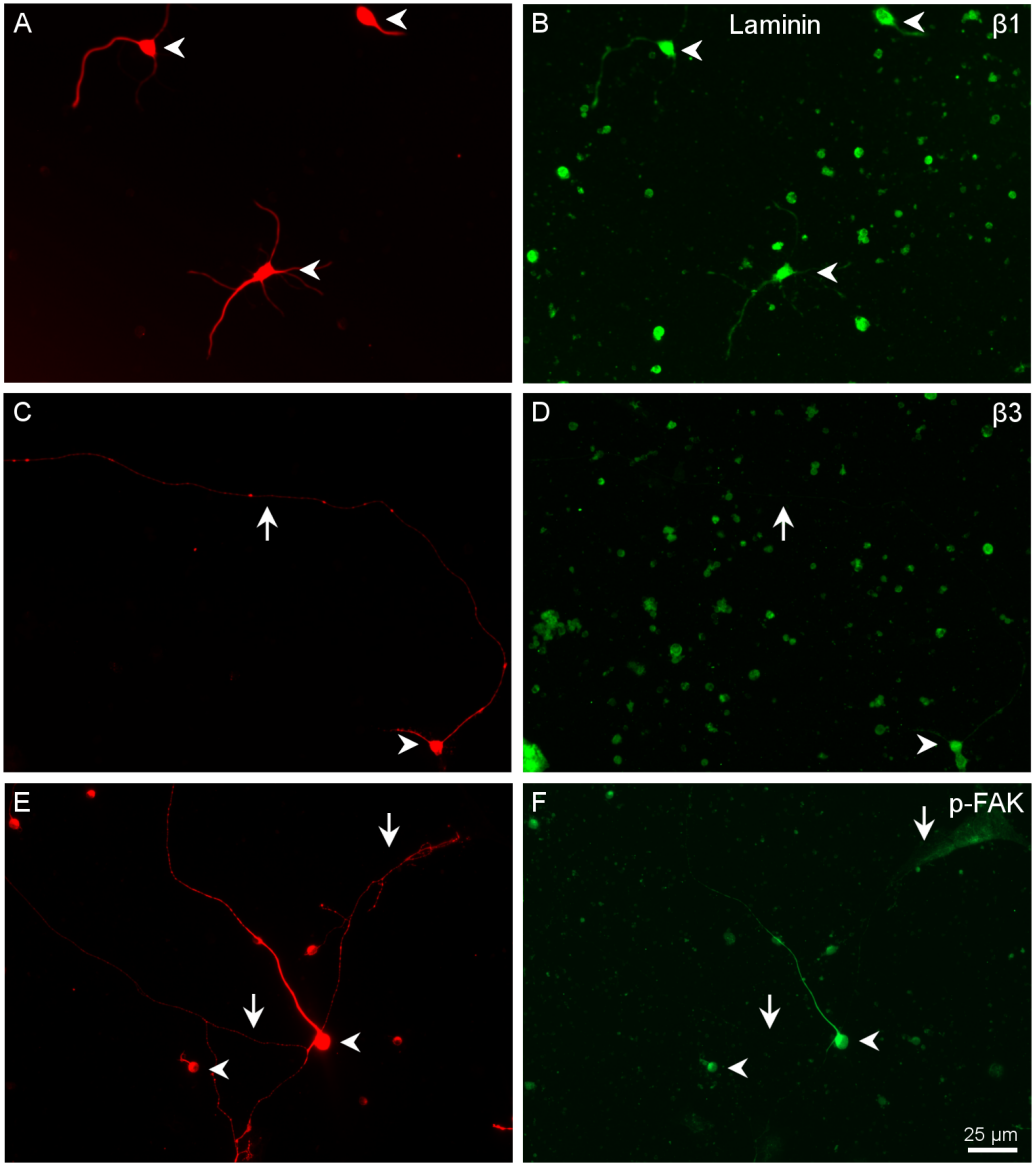
Distribution of β integrins and pY397 FAK in RGCs growing in laminin. Paired images A-B, C-D, and E-F of RGCs labelled with a βIII-tubulin antibody in red (A,C,E), and β1 (B) and β3 (D) integrins and pY397 FAK (F) in green. Note that the long RGC neurites pointed out with arrows in C and E are not labelled with either β3 or pY397 FAK while the rest of RGCs and their neurites expressed the integrins and were labelled for phosphorylated FAK. Scale bar for all pictures in F.

**Fig 6:**
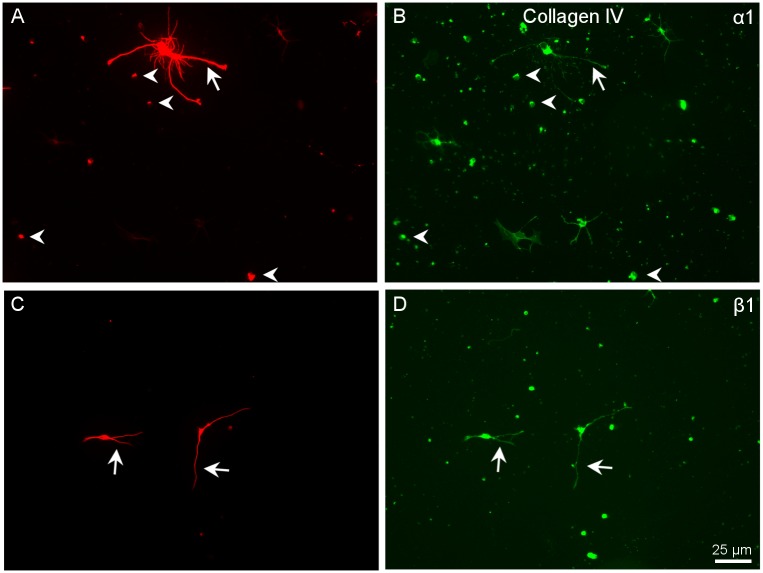
Distribution of α1 and β1 integrins in RGCs growing in collagen IV. Paired images A-B and C-D of RGCs labelled with a βIII-tubulin antibody (A,C) in red and for α1 (B) and α1 (D) integrins in green. Note that all RGC cell bodies (arrowheads) as well as the neurites (arrows) are labelled with both integrins. Scale bar for all pictures in D.

**Fig 7:**
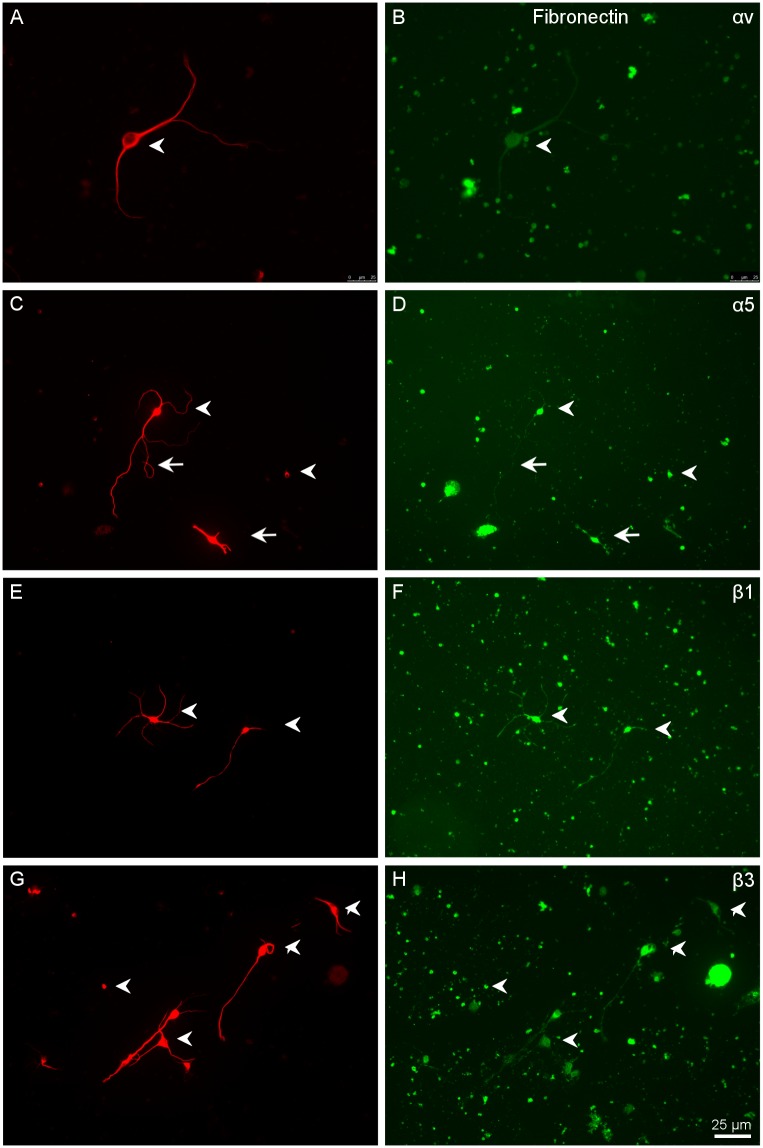
Distribution of αV, α5, β1 and β3 integrins in RGCs growing in fibronectin. Paired images A-B, C-D, E-F and G-H of RGCs labelled in red with a βIII-tubulin antibody (A, C, E, G) and αV (B), α5 (D), β1 (F) and β3 (H) integrins are visualised in green. Note that αV was not expressed in RGCs; only a very weak stain can be distinguished while the rest of integrins were expressed in the cell bodies (arrowheads) as well as in the neurites (arrows). Scale bar for all pictures in H.

Because the RGCs showed different levels of survival and process outgrowth on different substrates, we analysed the distribution of the potential binding integrins, αV, α1, α3, α5, β1 and β3 integrins. We found that in all analysed coverslips (at least 9 per substratum) RGCs expressed all the integrins detected within the cell bodies that we stained in retinal sections, with the exception of αV which was not seen in the cultured in RGCs. Overall, there was no correlation between RGC morphology, defined by parameters analysed previously (complexity or neurite length) and integrin expression or distribution. However, the staining intensity of some of the integrins was very low or only located within the cell body. In most cases the RGC soma had more intense immunoreactivity than the neurites (Figs 4, 5, 6, and 7). Furthermore, the main neurites were more evidently labelled than thin long neurites or secondary neurites (Figs 4 and 5). No quantification of the labelling was done. Nevertheless, comparative distribution between neighbouring cells gave us the difference in intensity between cells located in the same substratum. In order to confirm that the cells that we were observing were RGCs, double staining with βIII-tubulin was confirmed. In Figs 4, 5, 6, and 7 we have summarized the most representative expression of the different integrins in all the substrata studied.

RGCs growing on L expressed α1, α3 and α5 integrin (Fig 4). The integrin most intensely labelled was α1, especially in a subtype of RGCs with large cell bodies and long neurites. α3 and α5 also showed strong labelling in all RGC cell bodies and neurites (Fig 4). β1 was expressed in all RGCs without exception and was also present in the very long neurites (Fig 5). β3 was present in the cell body and the initial part of the neurites but not in long neurites (Fig 5C and 5D) From the immunohistochemistry results we can conclude that RGCs growing in L probably use either α3β1 or α5β1 to adhere to the substrate.

As we described before, cells growing in collagen tended to grow more neurites but they were relatively short and the growth cones were larger than in other substrata. We found very similar integrin expression in cells growing in CI and CIV with α1 and β1 being expressed in all cells and neurites. In addition, small cells, which looked like microglial cells, also expressed the α1 integrin subunit (Fig 6).

RGCs growing on F showed very faint labelling for αV while α5 integrin was expressed in all RGC cell bodies and neurites. Both β1 and β3 integrins were expressed in all RGC cell bodies and neurites. Thus, α5βI is a very likely integrin involved in RGC growth on F (Fig 7).

### Activation of the integrins *in vitro*, the phosphorylation of FAK

Where integrins bind to a ligand an adhesion complex may form which can be identified by the presence of the phosphorylated form of FAK. We therefore stained the cultures with an antibody to phosphorylated-tyrosine-397 FAK (pY397 FAK). We found positive staining in all RGCs examined. Very intense staining in the cell body and significant staining in all neurites was found. As in the case of the labelling for the α3, α5 and β1 integrin subunits, large diameter neurites showed more intense labelling than thinner ones. Cells that did not produce any neurite showed a very intense immunoreactivity for pY397FAK (Fig 5E and 5F).

## Discussion

### Subtypes of RGCs have different affinity for different ECM

In this study we have classified adult RGCs according to their capacity to grow and extend neurites on different substrates. We found that there are at least three types of cells depending on the ratios of growth and branching of their neurites: (1) RGCs that survive but do not grow any neurites (low level of complexity). These were found on all the substrates. These cells expressed all integrins tested in the present study and stained for pY397 FAK. These cells were more abundant on PL and L and scarcer on CI. (2) RGCs extending three or more neurites (high level of complexity) that grew moderate distances and were found particularly on collagen. (3) RGCs that grew a very long neurite, more than 5 times longer than the neurites of other RGCs, found chiefly on L. However, the long neurites from these (3) type of cells did not express any axonal markers.

### Poly-Lysine and laminin favoured the survival of RGCs

The number of cells that survived on PL and L after 6 days in culture was larger than in CI (Fig 1). Laminins are crucial to neurite outgrowth and to the structure of the neuronal synapse. They are also a major component of the ECM and basement membranes in the retina, being involved in RGC migration and subsequent development [20]. After dissociation of the retina, only a subpopulation of RGCs survives, and survival is dependent on cells attaching to their substrate in the first hours after. Loss of attachment to the matrix causes apoptosis in many cell types, this phenomenon, referred to as “anoikis” [21] may be the cause of death of the subpopulation of RGCs that were plated on CI. However, the RGCs that survived on collagen displayed more complexity (Fig 1). When we looked at the RGCs growing on collagens, we found that the neurites had prominent growth cones, especially the cells that had more neurites (complex cells) as compared to the growth cones of RGCs growing in other substrates (Fig 6).

Laminin as well as CI is present in the vitreous associated with the neuronal retina and synthesised by retinal astrocytes that are in direct contact with RGCs [22]. Elevation of CI expression in the RGC layer has been associated with the elevation of intraocular pressure [23]. CIV predominates in the vitreous base of the inner limiting membrane of the retina. Thus, adult RGCs are in contact with laminin, collagen I and IV as well as with fibronectin *in vivo*. However, *in vitro*, RGCs react differently to the different substrata as we have described in the present study. Since, *in vivo*, a combination of ECM is present in the inner limiting membrane; it is possible that different RGCs will have different affinities to different substrata depending of the location of the cells. *In vivo*, the cell bodies of some RGCs are in direct contact with astrocytes while others are more in contact with the membranes of the Müller cells [24]. Hence, it is possible that the influence of one or other components of the ECM that surrounds the RGCs could influence the expression of different integrins in the cell body. This would have a direct impact on the affinity to different substrates during the first moments after seeding the cells.

Laminin is important for RGC axon growth during development. In the adult its local distribution is in the inner limiting membranes, in contact with RGC axons [25]. A decrease in laminin expression has been associated with RGC death in insults like glaucoma [23] and optic nerve ligation [26]. Moreover, an important function of laminin surrounding the neuronal cell body and dendrites in regeneration has been suggested [27]. Furthermore, it has been demonstrated that axons grow predominantly on laminin, and the length of axons and the proportion of regenerating neurons varies with the concentration of laminin used for coating [28]. The use of different concentrations of laminin could explain why some studies conclude that RGCs lose the response to laminin with maturation [29] while we found that adult RGCs had a preference for laminin as a substrate.

Some RGCs that grew very long neurites; these were seen most frequently on laminin compared to the other substrata studied (Fig 1). The reason why these cells elaborated such long neurites compared to the rest of the RGCs is unknown. In previous studies on adult RGCs, we found that the elongation of neurites was favoured when cells grew in a confluent monolayer of retinal Müller glia (RMG) but was also promoted by RMG-conditioned medium as well as by addition of BDNF to the culture medium [14]. In the present study, we did not add any growth factors or conditioned medium to the culture media. No Müller cells were present in the cultures (data not shown), it is clear that laminin can promote the growth of these long neurites. One provocative hypothesis is that these fast growing cells are the RGCs, which, during development, cover longer distances from the peripheral retina to the most distal areas of the superior colliculus, and that they keep the memory for extending long neurites on laminin, as happens during development. Axons grow to the target centres in the brain along a stereotypic route, as if responding to cues distributed along the pathway. The distribution of laminin within the pathway is consistent with its localization at the end-feet of neuroepithelial cells that line the route, and it continues to be expressed at these marginal sites during early development. At later stages, laminin becomes restricted to the basal lamina at the retinal inner limiting membrane and the pial surface of the optic pathway [8]. It is known that central nervous system neurones may undergo a change in their substrate requirements for neurite outgrowth as they mature, but it is also possible that the shock of being placed into culture lead to re-expression of some of the molecules that were essential during development.

RGCs that do not grow any neurites (low complexity) expressed all the integrins that we analysed independently of the substratum. It is clear that for this specific RGC type the addition of some additional factors is needed to induce their regeneration; however, it is also clear that other cells growing very close to these do not have the same requirements because they grew long axons.

Although many studies have implicated bcl2 in promoting RGC survival, bcl-2 was recently reported to be an intrinsic genetic switch required for RGC axon regeneration [30]. Earlier studies had demonstrated that overexpression of bcl-2 does not promote regeneration of axotomized RGCs *in vivo* [31,32]. Thus, it has been proposed that the ability of bcl-2 to enhance the number of regenerating RGCs after injury is most likely due to its ability to promote the survival of RGCs that would normally die when injured and disconnected from their target-derived trophic signals [12]. It is well known that the number of cells able to survive after dissociation of an adult retina is very low. It is also possible that only the cells able to activate the bcl-2 mechanism can survive. Nonetheless, in the present study we have demonstrated that the subtype of cells able to survive contains cells with differing axon growth abilities. Thus, there are cells able to regenerate long neurites, other cells are able to produce multiple branches, while others just survive without growing any neurite at all. We can conclude that the internal information of the RGCs allows the cells to regenerate following different patterns and the addition of specific external factors is not needed. It is also possible that adult RGCs that are known to express neurotrophins and their receptors *in vivo* [33], as well as *in vitro* [13], will secrete their own growth factors acting through receptors present in the cell body in an autocrine manner since it has been demonstrated that RGCs canincrease their BDNF expression after damage [34]. At least for BDNF and its receptor TrkB, we have previously demonstrated that they are colocalized in the cell body of all RGCs [35].

### Expression of different integrins by the RGCs

We asked whether the presence of different substrates could affect the production of different integrins by RGCs. We found that independent of the substrate all the integrins studied were expressed by the RGCs, although with different intensities.

The four ECM molecules that we studied are expressed *in vivo* in the visual pathway. We found that, while laminin and fibronectin and to a lesser extend collagens were mainly present in the adult outer limiting membrane, collagens were also present in the optic nerve (Fig 2). Thus, RGCs *in vivo* are in direct contact with the 4 ECM molecules. However, the subcellular contact is different. While RGC somas are located in the retina in contact with (1) laminin secreted by astrocytes and Müller glia, (2) laminin from the Müller end feet and (3) collagen present in the vitreous fibrils. On the other hand, the RGC axons are more in direct contact with collagen I and IV from the optic nerve (Fig 2). When we looked at the expression of our candidate α and β integrin subunits, we found that all of them were expressed at different levels by the RGCs *in vivo*. However, β3 was present mainly in the inner plexiform layer (Fig 3), indicating its likely role in modulating information within other cells within the retina. The rest of the integrins were more associated with the RGC layer and outer limiting membranes (Fig 3). Similar to what we found for the neurotrophins and their receptors [13], in the present study, we showed that RGCs *in vitro* expressed the same integrins as *in vivo*.

*In vitro*, α1 was very weakly expressed in the RGC cell bodies and absent in the neurites except for a very small number of RGCs that were labelled much more brightly for this integrin (Fig 4). These cells had large cell bodies and at least three neurites. α3, α5 and β1 integrins were more intensely labelled in the cell bodies and short neurites. In the long neurites, α3 had very weak stainning, while α5 was more intense (Fig 4). β1 was detected in all cell bodies and in short neurites and more weakly in long neurites (Fig 5). This indicates that the integrin most likely involved in the growth of RGCs on laminin is α5β1 and to a lesser extend α3β1. For some cells, α1β1 might also play a role in either the initial attachment or the extension of neurites.

In order to prove that the integrins were activated we looked for the presence of pY397 FAK. FAK was phosphorylated in all types of RGCs. However, in those cells that extended long neurites, these long processes had less pY397 FAK. This could be due to the smaller diameter of these neurites, and it was thus more difficult to detect the label (Fig 5). Therefore, we conclude that RGCs growing in laminin have activated integrins present, which signal through the FAK route.

Analysing the expression of integrin subunits in RGCs growing on collagen, it seemed that α1β1 might be the integrin combination used for the interaction with this substratum. In addition to RGCs, few other cells were present in the cultures that also expressed the α1 integrin subunit (Fig 6).

On fibronectin, RGCs expressed only low levels of the αV integrin subunit. However, α5, β1 and β3 were present in the cell bodies as well as in the neurites with moderate intensity. This indicates that α5β1 was the most likely combination of integrins expressed by RGCs when growing on fibronectin. Nevertheless, in an *in vivo* situation, several ECM molecules are present in the same areas, and integrins might work in concert to ensure proper signalling in the environment. As has been shown in other cell types, integrins exist in different conformations; and these appear to reflect different ligand-binding capabilities. For example, it has been found that endogenous α2β1 on melanoma cells, but not on platelets, binds to laminin, whereas both cells use α2β1 to bind to collagen [36]. Moreover, different cell types transfected with the same integrin subunit recognized different substrata [37]. These results suggest that the cellular environment can determine the ligand-binding specificity via conformational changes.

## Conclusions

In the present study we were able to show that adult rat RGCs can survive and grow in the presence of different ECM environments (laminin, collagen I, collagen IV, and fibronectin). All RGC types were lablelled by antibodies to all the integrins that we stained for in the cell body but varying intensity. Some cells did not express the integrins in the neurites, especially not in the long ones. The integrins were activated and signalled through the FAK pathway. Thus, in future *in vitro* experiments, in which the regenerative capacity or sensitivity to different drugs will be studied, it will be important to pay attention to the diversity of the RGCs. Further studies should be done to elucidate the different molecular characteristics of the RGCs subtypes in order to understand the possible different sensitivity of different RGCs to damage in diseases like glaucoma in which not all RGCs die at the same time.
